# Examining the Relation Between Prenatal Emotion Dysregulation and Toddler Vocabulary Development: A Biobehavioral Approach

**DOI:** 10.1002/dev.70018

**Published:** 2025-01-09

**Authors:** Kira R. Wright, Madeleine Bruce, Anna M. Zhou, Sarah E. Maylott, K. Lee Raby, Elisabeth Conradt, Sheila E. Crowell

**Affiliations:** ^1^ Department of Psychology The University of Utah Salt Lake City Utah USA; ^2^ Department of Psychiatry and Behavioral Sciences Duke University School of Medicine Durham North Carolina USA; ^3^ Department of Psychiatry University of Colorado Anschutz Medical Campus Aurora Colorado USA; ^4^ Department of Psychology University of Denver Denver Colorado USA; ^5^ Department of Psychology University of Oregon Eugene Oregon USA

**Keywords:** emotion dysregulation, longitudinal, maternal stress, pregnancy, respiratory sinus arrhythmia, toddler vocabulary

## Abstract

Early language is shaped by parent–child interactions and has been examined in relation to maternal psychopathology and parenting stress. Minimal work has examined the relation between maternal emotion dysregulation and toddler vocabulary development. This longitudinal study examined associations between maternal emotion dysregulation prenatally, maternal everyday stress at 7 months postpartum, and toddler vocabulary at 18 months. Data were collected from 289 typically developing, monolingual children (54% female) and their mothers (63% White and non‐Hispanic; 56% held a college degree). During pregnancy, maternal emotion dysregulation was measured via self‐report and resting respiratory sinus arrhythmia (RSA). Mothers completed questionnaires about their perceived everyday stress and their child's vocabulary at 7 and 18 months postpartum, respectively. Path analysis revealed that expectant mothers’ self‐reported emotion dysregulation was indirectly associated with toddlers’ expressive vocabulary via their level of postpartum perceived everyday stress. In addition, prenatal maternal resting RSA directly predicted toddlers’ expressive vocabulary size. These findings yield insights into the mechanisms by which perinatal mental health may shape early language development and highlight the potential utility of interventions targeting emotion dysregulation during pregnancy.

## 1 Introduction

Language emerges early in infancy, is highly modifiable, and is a fundamental skill that sets the stage for various developmental outcomes. For example, language is an essential tool that enables young children to inhibit impulses, regulate strong emotions, and engage in prosocial interactions with peers (Bruce, Ermanni, and Bell 2023; Petersen et al. 2013; Yew and O'Kearney 2013). Vocabulary development is rapid as children begin comprehending words (receptive language) around 9 months and producing their first words (expressive language) near the end of the first postnatal year (Fenson et al. 1994). Grounded in the sociocultural theory of development (Vygotsky 1962), there is clear evidence that parent–child interactions shape language acquisition (Kuhl 2007). Young children pay close attention to speech sounds in their environment, especially when they are produced by warm and responsive caregivers in the form of short utterances, variable prosody, and a slow speaking rate (i.e., infant‐directed speech; Fernald et al. 1989). However, considerable variability has been observed in the quantity and quality of maternal input during toddlerhood (Hart and Risley 1995; Huttenlocher et al. 2010), which may in part be due to maternal mental health factors. Indeed, various studies have found significant associations between maternal psychopathology symptoms and smaller vocabularies during toddlerhood (Bornstein, Henry, and Manian 2021; O'Leary et al. 2019; A. M. Rogers et al. 2023). Thus, identifying maternal predictors of early vocabulary acquisition may yield novel insights into the contextual factors that shape language learning, which in turn can inform prevention and intervention efforts.

One source of variability in mother–child interactions appears to be maternal mental health. For instance, mothers experiencing depressive symptoms speak less to their toddler and produce less infant‐directed speech during parent–child interactions (Lam‐Cassettari and Kohlhoff 2020; Severo et al. 2023). However, developmental studies have often focused on diagnostic categories of psychopathology (e.g., depression, anxiety) as opposed to broader aspects of maternal functioning, such as regulation of emotions. This represents a core knowledge gap as this emphasis on clinical diagnosis limits our understanding of the transdiagnostic and psychobiological markers of maternal mental health, which may relate to toddler cognition and language development. One factor underlying several categories of psychopathology is emotion dysregulation (Cole and Hall 2008), although its relation to language acquisition remains poorly understood.

Furthermore, few studies have investigated potential mechanisms underlying relations between maternal mental health and language development. Multiple studies have identified links between maternal stress and mental health (Feng, Xu, and Lei 2023; Hammen 1991; Hammen et al. 2009; Levine, Milyavskaya, and Zuroff 2020), as well as associations between maternal stress and children's language development (Pierce, Reilly, and Nelson 2021; Spinelli et al. 2022). Therefore, it stands to reason that maternal everyday stress may be a potential behavioral mechanism underlying associations between maternal mental health and child language development. The developmental cascade framework posits that early experiences and competencies interact over time to shape children's subsequent developmental pathways (Masten and Cicchetti 2010). Accordingly, maternal emotional difficulties may exert a downstream effect on language development by shaping aspects of the early caregiving environment (e.g., maternal stress). Thus, the overarching purpose of this study was to examine longitudinal relations between prenatal maternal emotion dysregulation, postnatal maternal everyday stress, and toddler vocabulary acquisition.

### 1.1 Toddler Vocabulary Development

Language emerges in the context of warm, engaging, and reciprocal interactions with mature linguistic speakers (Feldman 2019). In other words, children rely on caregivers to expose them to lexically rich and varied speech in a manner that is readily comprehended early in development (i.e., via infant‐directed speech; Golinkoff et al. 2015). Children's early speech preference is thought to cue them into the statistical properties of their native language(s) during parent–child interactions (Saffran 2020; Vouloumanos et al. 2010). Saffran (2020) reported that preverbal children are able to track linguistic patterns from their caregivers, which in turn allows them to develop expectations about what should happen next in their environment. Furthermore, Tsao, Liu, and Kuhl (2004) provide evidence that speech perception in early infancy predicted language performance in toddlerhood, indicating the importance of early language experiences.

Across the first postnatal year, many children start producing speech sounds and communicative gestures, which is followed by more complex word learning and meaningful speech production (Fenson et al. 1994; 2007). Expressive and receptive vocabulary development are fundamental components of early language acquisition, and they are heavily influenced by dynamic toddler–caregiver interactions. For instance, Huttenlocher et al. (2010) found that more variable linguistic input in infancy predicts faster language growth in early childhood, while Bruce, Ermanni, and Bell (2023) reported that higher maternal positive affect at 5 months predicted greater vocabulary skills at 24 months. Caregivers often use infant‐directed speech when speaking with their child as it captivates toddlers’ attention and cues them into the linguistic properties of their native language, thereby facilitating early language learning (Fernald et al. 1989; Golinkoff et al. 2015; Weisleder and Fernald 2013). For example, Weisleder and Fernald (2013) found that toddlers exposed to infant‐directed speech had a larger expressive vocabulary and more efficient speech‐processing skills at 24 months. This means that the quality of maternal speech is as important as the quantity of input for toddler language acquisition, and individual differences in maternal speech composition, lexical diversity, and speech quantity are predictive of toddler language outcomes (Huttenlocher et al. 1991; Huttenlocher et al. 2010). Additionally, higher levels of maternal responsiveness and warmth during parent–child interactions are associated with greater language gains in toddlerhood and childhood (Leigh, Nievar, and Nathans 2011; Steelman et al. 2002). Thus, nurturing and responsive caregiving behaviors, coupled with lexically rich maternal input, play an important role in promoting toddler vocabulary development.

### 1.2 Maternal Mental Health and Toddler Outcomes

Maternal psychopathology during the perinatal period has been shown to predict several early developmental outcomes, including language development. For example, Bornstein, Henry, and Manian (2021) found that mothers diagnosed with clinical depression had children with lower receptive and expressive language abilities at 15 and 24 months in comparison to children with nondepressed mothers. Even when mothers experienced a remission of depression later, exposure to early maternal depressive symptoms was linked to lower language comprehension and production in toddlerhood (Bornstein, Henry, and Manian 2021). Additionally, A. M. Rogers et al. (2023) reported associations between greater antenatal anxiety and poorer toddler language development. Thus, maternal internalizing psychopathology is prospectively linked to toddler language acquisition. Severo et al. (2023) theorized that difficulties engaging in and maintaining joint attention during dyadic interactions may account for the association between maternal depressive symptoms and early language development outcomes. Indeed, maternal depressive symptoms are associated with less infant‐directed speech, fewer verbal interactions, and less contingent and timely responses to their child's cues (Bernard et al. 2018; Borairi et al. 2024; Lam‐Cassettari and Kohlhoff 2020; A. Rogers et al. 2020; Severo et al. 2023).

Far fewer studies have examined links between *prenatal* maternal mental health and early vocabulary development, although the available literature reports similar developmental patterns (e.g., O'Connor et al. 2002; O'Leary et al. 2019). For instance, O'Leary et al. (2019) report that expectant mothers with clinical depression had children with lower expressive and receptive language abilities at 12 months, although these authors were unable to establish a mechanism underlying this association. What is clear from previous research is that children of mothers with perinatal depression are at an increased risk of exhibiting poor language outcomes, yet additional research conducted during prenatal development is needed. Furthermore, existing developmental studies have often focused on broad diagnostic categories as opposed to mothers’ underlying and transdiagnostic biobehavioral functioning (e.g., emotion dysregulation). This is a noteworthy limitation of the current literature as the traditional diagnostic model tends to minimize the complexity, dimensionality, and comorbidity of mental health problems (Dalgleish et al. 2020). In sum, research examining transdiagnostic markers of maternal emotion dysregulation during prenatal development is needed to better understand associations between maternal mental health and toddler vocabulary acquisition.

### 1.3 Emotion Dysregulation: A Biobehavioral Approach

Emotion regulation refers to the maintenance and/or modulation of an emotional response to achieve one's goals (Beauchaine 2015; Thompson 1994). Adaptive emotion regulation is related to better emotional functioning, social interactions, and overall well‐being (Gross and John 2003). In contrast, emotion dysregulation is characterized by difficulty in monitoring, evaluating, and/or modifying emotional responses to accomplish an individual's emotional goals (Gratz and Roemer 2004). Consistent with the Research Domain Criteria (RDoC) framework, emotion dysregulation is widely considered to be a transdiagnostic factor underlying risk for diverse forms of adolescent and adult psychopathology (i.e., depression, anxiety, bipolar disorder, and borderline personality disorder; Cole and Hall 2008; Cuthbert 2020; Fernandez et al. 2016). For example, McLaughlin et al. (2011) report that emotion dysregulation was a prospective risk factor for adolescent psychopathology, although they found no evidence of an inverse association (i.e., psychopathology predicting emotion dysregulation). Because emotion dysregulation influences adults’ social functioning and self‐regulatory abilities (Gross and Munoz 1995), it may be the case that maternal emotion dysregulation has a downstream effect on child development. Yet, there is surprisingly little research examining the association between prenatal maternal emotion dysregulation and child outcomes, and much of the existing work has focused on child socioemotional development (e.g., Ostlund et al. 2019).

#### 1.3.1 Capturing Maternal Emotion Dysregulation: Self‐Report Versus Physiological Indices

One common measure of maternal emotion dysregulation is the Difficulties in Emotion Regulation Scale (DERS; Gratz and Roemer 2004), which is an empirically supported, multidimensional conceptualization of self‐reported emotion regulation difficulties. A handful of studies have investigated whether maternal DERS scores are associated with early childhood outcomes. For example, Ostlund et al. (2019) reported that newborns exposed to high levels of self‐reported maternal emotion dysregulation in utero had lower levels of arousal and attentional capabilities at birth, both of which have been linked to children's later behavioral, emotional, and cognitive outcomes (Liu et al. 2010). Furthermore, Binion and Zalewski (2018) found that preschool‐age children of emotionally dysregulated mothers (as indicated by the DERS) use less effective regulation strategies in comparison to children of more regulated mothers. Additionally, Leerkes, Su, and Sommers (2020) found that mothers scoring high in self‐reported emotional dysregulation reported their toddlers as having more problem behaviors. Thus, maternal emotion dysregulation, as captured by the DERS, appears to relate to young children's attentional, behavioral, and self‐regulation capabilities.

Physiological indices during rest can serve as a biomarker of individual differences in maternal emotion regulation capabilities (Beauchaine and Thayer 2015; Beauchaine 2015). Respiratory sinus arrhythmia (RSA) refers to the natural variation in heart rate across the respiratory cycle and is captured by the high‐frequency heart rate variability that coincides with respiration (Beauchaine and Thayer 2015). RSA is an index of parasympathetic‐linked cardiac activity, specifically the influence of the vagal cranial nerve on heart rate variability (Beauchaine 2015). At rest, there is typically greater beat‐to‐beat variability in heart rate (i.e., higher RSA), compared to periods of high stress, during which there is frequently less beat‐to‐beat variability (i.e., lower RSA) due to a withdrawal in parasympathetic influence through the vagus nerve on heart rate (Beauchaine 2015). Consequently, resting RSA is thought to reflect one's ability to adapt to environmental demands (Beauchaine 2015; Price and Crowell 2016; Yaroslavsky et al. 2013). Individuals with higher resting RSA are often able to modulate their cardiac activity more efficiently and regulate their emotional arousal (Beauchaine 2015; Porges 2007). In contrast, low resting RSA is often associated with increased vulnerability to psychopathology and emotion dysregulation (Beauchaine 2015; Beauchaine et al. 2019). Taken together, capturing expectant mothers’ emotion dysregulation via biological (i.e., resting RSA) and self‐report methods (i.e., DERS) allows for a biobehavioral measurement approach that better encompasses the multifaceted nature of this transdiagnostic construct as it may relate to toddler vocabulary development.

### 1.4 Maternal Everyday Stress

Given existing findings that maternal psychopathology is associated with toddler vocabulary, it is likely that prenatal maternal emotion dysregulation will also predict early language outcomes. However, it is unclear whether specific postnatal contextual factors may account for this association. Perceived everyday stress can be defined as the subjective appraisal of stressors that is influenced by social support, coping mechanisms, or locus of control (Cohen, Kamarck, and Mermelstein 1983), and it has emerged as both an antecedent and consequence of poor mental health (Feng, Xu, and Lei 2023; Hammen 1991; Hammen et al. 2009; Levine, Milyavskaya, and Zuroff 2020). According to the stress generation hypothesis (Hammen 1991), individuals experiencing psychological distress may inadvertently behave in ways that generate stressful environmental conditions, which can in turn contribute to higher feelings of stress and depressive symptoms. It has also been theorized that experiencing negative emotions might deplete the cognitive resources needed to cope with environmental demands, thereby leading to greater subsequent stress levels (Feng, Xu, and Lei 2023; Padmala, Bauer, and Pessoa 2011). For example, among youth aged 8–17, greater emotion dysregulation predicted a greater number and frequency of stressful events measured over the next year and a half (Schneider et al. 2021). Furthermore, Shih and Eberhart (2008) demonstrated that adult women's subclinical symptoms of depression were associated with greater interpersonal stress levels. In sum, higher maternal emotion dysregulation during pregnancy may similarly predict high levels of postnatal maternal everyday stress, although to our knowledge this association has yet to be empirically examined.

There is also evidence to suggest that maternal parenting stress predicts both the quality and quantity of child–caregiver interactions. For example, Spinelli et al. (2022) showed that caregivers reporting higher perceived parenting stress spoke less to their young children and used a more complex form of infant‐directed speech, one that is typically characteristic of interactions with adults and older children. Furthermore, mothers who reported higher caregiving stress levels had children who produced fewer vocalizations and participated in less conversational turns at 6 and 12 months (Pierce, Reilly, and Nelson 2021; see also Noel, Peterson, and Jesso 2008). To date, many studies have focused narrowly on individual dimensions of maternal everyday stress (i.e., parenting stress) in relation to child language development. However, everyday stress is multifaceted, stems from several interrelated sources, and consists of individual variation in the appraisal of the same stressors (Evans and English 2002; Troller‐Renfree et al. 2022). As evidenced by the Family Stress Model (Conger and Conger 2002), an array of everyday stressors, including interpersonal conflict, parenting concerns, and financial hardship, have been linked to adverse outcomes in early childhood (Craft, Perry‐Jenkins, and Newkirk 2021; Neppl, Senia, and Donnellan 2016; Noble, McCandliss, and Farah 2007). Thus, the current study extends previous developmental research by investigating whether mothers’ postnatal everyday stress levels account for our hypothesized association between prenatal maternal emotion dysregulation and toddler language outcomes.

### 1.5 Current Study

The overarching purpose of this longitudinal study was to examine relations between biobehavioral measures of maternal emotion dysregulation (prenatally), maternal everyday stress (7 months), and toddler expressive/receptive vocabulary (18 months). Previous research has uncovered associations between maternal mental health (e.g., symptoms of depression) and child language; however, no studies to date have investigated the predictive validity of biobehavioral measures of maternal emotional dysregulation in relation to toddler vocabulary acquisition. This existing emphasis on psychiatric diagnoses obscures our understanding of whether and how the biobehavioral processes that underlie many clinical disorders may influence toddler development, which makes conducting replication research and developing targeted interventions all the more challenging (Cuthbert and Insel 2013). Furthermore, the developmental pathway(s) by which maternal psychopathology influences young children's language development remain poorly understood. Since perceived stress is associated with maternal depressive symptoms and children's language development, it stands to reason that mothers’ self‐reported level of everyday stress may partially account for the anticipated association between maternal emotion dysregulation and children's vocabulary development.

To this end, we developed two primary research questions: First, is expectant mothers’ emotion dysregulation directly associated with toddler vocabulary size at 18 months? Considering previous research linking maternal depression to language development (e.g., Bornstein, Henry, and Manian 2021), we hypothesized that mothers with higher levels of emotion dysregulation during pregnancy (i.e., higher DERS and lower resting RSA) would have toddlers with smaller receptive and expressive vocabularies. Our second research question was: What role does maternal everyday stress at 7 months play in the hypothesized relation between prenatal emotion dysregulation and toddler vocabulary at 18 months? We hypothesized that mothers with higher levels of emotion dysregulation during pregnancy (i.e., higher DERS and lower resting RSA) would report higher levels of everyday stress when their child was 7 months old. In turn, we also predicted that mothers who report greater everyday stress during their child's first postnatal year would have toddlers with smaller expressive and receptive vocabularies the following year. Given this pattern of hypothesized direct effects, we expected to find an indirect effect between maternal emotion dysregulation and toddler vocabulary at 18 months in accordance with the developmental cascade framework. In sum, the current study incorporates biobehavioral measures of emotion dysregulation and considers both pre‐ and postnatal maternal factors in relation to toddler vocabulary development. This integrative approach may provide a more nuanced understanding of these interconnected constructs, enabling us to address previously unanswered questions about the downstream effects of perinatal maternal factors on early vocabulary acquisition.

## 2 Method

### 2.1 Participants

Participants include 289 mothers and their typically developing, English‐learning children (54% assigned female at birth). Mother–toddler dyads come from a larger longitudinal study (*N* = 385) examining intergenerational transmission of emotion dysregulation from the third trimester to 36 months postpartum. Pregnant individuals were recruited from the local community during prenatal appointments at University of Utah OB/GYN clinics, via email, flyers, and brochures from January 2016 to January 2023 in two cohorts. Pregnant individuals interested in participating in the study completed a screener that consisted of the DERS and eligibility criteria questions (i.e., ages 18–40, no pregnancy complications such as preeclampsia or gestational diabetes, no substance use, and anticipated singleton delivery). The majority of participants identified as White and non‐Hispanic (62.6%), approximately a fourth (26.6%) reported a $50,000–$79,999 household income, and a third (33.2%) of the sample are college graduates. In addition to recruiting a socioeconomically diverse sample, the study oversampled individuals with high and low emotion dysregulation to achieve a uniform distribution of DERS scores. Additional demographic information for the sample was collected during the prenatal time point and is presented in Table 1.

**TABLE 1:** Participant demographics.

Demographic variable	*N*	Percent	Mean (SD)	Min–max
Maternal age in years (at prenatal visit)	288	99.7%	29.32 (4.77)	18–40
Maternal race and ethnicity	288	99.7%		
American Indian or Alaskan Native and not Hispanic/Latina	5	1.7%		
American Indian or Alaskan Native and Hispanic/Latina	1	0.3%		
Asian and not Hispanic/Latina	19	6.6%		
Native Hawaiian or Other Pacific Islander and not Hispanic/Latina	4	1.4%		
Black or African American and not Hispanic/Latina	7	2.4%		
Black or African American and Hispanic/Latina	1	0.3%		
White and not Hispanic/Latina	181	62.6%		
White and Hispanic/Latina	37	12.8%		
Prefer to self‐report and not Hispanic/Latina	7	2.4%		
Prefer to self‐report and Hispanic/Latina	6	2.1%		
Multiracial and not Hispanic/Latina	11	3.8%		
Multiracial and Hispanic/Latina	2	0.7%		
Decline to answer race and Hispanic/Latina	2	0.7%		
Decline to answer race and not Hispanic/Latina	1	0.3%		
White and decline to answer ethnicity	2	0.7%		
Multiracial and decline to answer ethnicity	1	0.3%		
Decline to answer race and ethnicity	1	0.3%		
Maternal education	286	99.0%		
Less than 12^th^ grade	8	2.8%		
High school graduate or equivalent	29	10.0%		
Some college or technical school	87	30.1%		
College graduate	96	33.2%		
Any postgraduate school	66	22.8%		
Household income	262	90.7%		
Under $9000	12	4.2%		
$9000–$14,000	10	3.5%		
$15,000–$19,999	7	2.4%		
$20,000–$24,999	6	2.1%		
$25,000–$29,999	14	4.8%		
$30,000–$39,999	22	7.6%		
$40,000–$49,999	16	5.5%		
$50,000–$79,999	77	26.6%		
$80,000–$99,999	36	12.5%		
$100,000 or more	54	18.7%		
Refuse to answer	1	0.3%		
Not sure	7	2.4%		
Maternal parity	270	93.4%	1.99 (1.04)	1–6
Maternal relationship status	286	99%		
Married	239	82.7%		
Not married	36	12.5%		
Separated or divorced	11	3.8%		
Child sex	289	100%		
Female	156	54.0%		
Male	133	46.0%		
Child race and ethnicity	288	99.7%		
American Indian or Alaskan Native and not Hispanic/Latina	1	0.3%		
Asian and not Hispanic/Latina	5	1.7%		
Asian and Hispanic/Latina	1	0.3%		
Native Hawaiian or Other Pacific Islander and not Hispanic/Latina	3	1.0%		
Black or African American and not Hispanic/Latina	7	2.4%		
White and not Hispanic/Latina	161	55.7%		
White and Hispanic/Latina	44	15.2%		
Prefer to self‐report and not Hispanic/Latina	2	0.7%		
Prefer to self‐report and Hispanic/Latina	1	0.3%		
Multiracial and not Hispanic/Latina	41	14.2%		
Multiracial and Hispanic/Latina	12	4.2%		
Decline to answer race and Hispanic/Latina	2	0.7%		
Decline to answer race and not Hispanic/Latina	3	1.0%		
White and decline to answer ethnicity	4	1.4%		
Multiracial and decline to answer ethnicity	1	0.3%		
Child gestational age in days (at delivery)	286	99.0%	274.46 (7.87)	239–290
Child birth weight in grams	282	97.6%	3349.8 (455.86)	1770–4560

Children from the larger longitudinal study who were too young to participate in the 18‐month time point were considered ineligible for the current study, and these mother–toddler dyads were excluded from our analyses (*N* = 37). An additional 59 dyads were excluded from this study because the child was multilingual learning (*N* = 35), was being monitored for a developmental delay (*N* = 17), was identified as having a hearing or vision impairment (*N* = 1), or met a combination of these reasons (*N* = 6). Multilingual children were excluded from our analyses because best practices indicate that these children's vocabulary size should be measured across each language they are acquiring (Byers‐Heinlein 2013), and only the English version of the language questionnaire was administered in the larger study. Previous work demonstrates that children with audio/visual impairments as well as developmental delays often exhibit atypical language acquisition patterns for reasons other than those that were a focus of this study (Meinzen‐Derr et al. 2018; Mosca, Kritzinger, and van der Linde 2015; Valla et al. 2015). Consequently, these children were also excluded from our analyses. Among our remaining sample of 289 mother–toddler dyads, 288 expectant mothers completed the DERS and 270 expectant mothers provided usable resting RSA data. At the postnatal time points, 219 mothers completed the everyday stress questionnaire when their child was 7 months and 187 mothers completed the language questionnaire when their child was 18 months. Mothers who did not complete the 7 or 18‐month questionnaires did not significantly differ from those who did as a function of maternal age, maternal race, household income, or child sex. However, mothers who did not possess a 4‐year college degree at the prenatal time point were more likely to not complete the 7‐month (*χ* ^2^ = 5.70, *df* = 1, *p* = 0.02) and 18‐month questionnaires (*χ* ^2^ = 6.50, *df* = 1, *p* = 0.01). As noted below, full information maximum likelihood was used to handle missing data, and maternal education was included as a covariate in the focal analyses.

### 2.2 Procedure

Study procedures were approved by the Institutional Review Board at The University of Utah. Upon consenting to participate in the larger longitudinal study, eligible participants completed the DERS and a demographics questionnaire using an online link. At the prenatal time point, expectant mothers participated in several behavioral tasks in the lab or in their home during which physiological data were collected (for additional details regarding remote research visits during the COVID‐19 pandemic, see Gao et al. 2022). Of interest in the current study, resting RSA was collected during a 10‐min rest period using heart rate. Prior to completing the 7 and 18 month in lab or remote research visits, mothers completed online questionnaires about themselves (e.g., the Everyday Stress Index [ESI]) and their child (e.g., the MacArthur–Bates Communicative Development Inventory).

### 2.3 Measures

#### 2.3.1 Maternal DERS (Prenatal)

The DERS (Gratz and Roemer 2004) was administered to measure prenatal maternal emotion dysregulation. The DERS consists of 36 items and assesses six aspects of emotion dysregulation: nonacceptance of emotional responses, difficulty in goal‐directed behavior, difficulties with impulse control, lack of emotional awareness, lack of emotional clarity, and limited emotion regulation strategies. The DERS uses a 5‐point Likert scale (1 = *almost never*, 5 = *almost always*), and sample items include “I pay attention to how I feel” and “When I'm upset it takes me a long time to feel better.” Participant responses were summed to generate an overall emotion dysregulation score, where larger values represent greater levels of emotion dysregulation. The DERS demonstrated strong internal consistency for the current sample at the prenatal time point (Cronbach's *α* = 0.96).

#### 2.3.2 Maternal Resting RSA (Prenatal)

Maternal electrocardiogram (ECG) data were collected during the prenatal lab visit using a standard three lead spot configuration and Mindware mobile devices (Mindware Technologies Ltd.). In the lab, trained research assistants placed electrodes on the participants’ chest and abdomen based on a three‐lead configuration. During the COVID‐19 pandemic, remote research sessions were conducted in the participants’ home, and participants placed the electrodes themselves while being guided by a trained research assistant on Zoom (see Gao et al. [2022] for remote research visit protocol). Mindware Technologies HRV 3.1.5 software was used to collect and process ECG data. RSA data were extracted from ECG R‐peaks in 60‐s epochs for a total of 10 min, during which the mother was instructed to relax and not engage in any activities to establish a baseline. RSA was processed as the beat‐to‐beat variability in ECG R‐peaks in the high‐frequency band of 0.24–0.60 Hz due to high respiration levels observed. Resting RSA was calculated as the average RSA across the 10‐min baseline task.

#### 2.3.3 Maternal Everyday Stress (7 Months)

The ESI (Hall, Williams, and Greenberg 1985) was administered to measure perceived maternal everyday stress at the 7‐month time point (approximately midway through the child's first postnatal year). The ESI consists of 20 items that examine role overload, financial concerns, parenting worries, employment problems, and interpersonal conflict and are rated on a 4‐point Likert scale (0 = *not at all bothered*, 3 = *bothered a great deal*). Sample items include “Having too many responsibilities,” “Owing money/getting credit,” and “Problems with your child(ren)’s behavior.” A cumulative everyday stress score was computed by summing the item responses such that larger values represent a higher level of perceived everyday stress at 7 months postpartum. The ESI has demonstrated good internal consistency within our sample (Cronbach's *α* = 0.83).

#### 2.3.4 Toddler Vocabulary (18 Months)

Mothers’ report of their toddlers’ early receptive and expressive vocabulary was assessed using the MacArthur–Bates Communicative Development Inventory: Words and Gestures (MCDI‐WG; Fenson et al. 2007) at the 18‐month time point. The MCDI‐WG is normed for 8‐ to 18‐month‐old children and consists of an 89‐item checklist measuring emerging receptive and expressive vocabulary. Example items from this measure include “Does your child understand and/or say: Stroller, Mouse, Ouch.” Cumulative scores are computed to determine both *receptive* and *expressive* vocabulary in which higher scores indicate higher vocabulary skills. The MCDI‐WG has demonstrated high internal consistency for our sample (Cronbach's *α* = 0.98), as well as good reliability and validity in previous research (Fenson et al. 2007).

### 2.4 Analytic Plan

Descriptive statistics and correlations were examined using SPSS (Version 26; Table 2). Data were then inspected for normality of distributions and outliers (values ±3 SDs). Path analysis was conducted in Mplus (Version 8; Muthen and Muthen 2012) to test hypothesized relations between maternal prenatal emotion dysregulation, postnatal everyday stress, and toddler vocabulary. Child sex assigned at birth (0 = *girl*; 1 = *boy*), child birth weight, and prenatal maternal education (0 = *no college education*; 1 = *college education*) were included as covariates in the main analyses, given established links to early language acquisition in the developmental literature (Hoff 2003; Madigan et al. 2015; Rice and Hoffman 2015). Maternal education was used as an index of socioeconomic status because previous research shows that education‐related disparities in vocabulary development persist even within low‐income populations (Justice et al. 2020; Fernald, Marchman, and Weisleder 2013). Full information maximum likelihood was used to handle missing data. This estimation method has been shown to produce less biased estimates and standard errors in comparison to other missing data techniques (e.g., pairwise or listwise deletion; Enders 2001). Bootstrapping was applied (*N* = 10,000), and the 95% confidence intervals (CIs) were calculated. An indirect effect is interpreted as statistically significant when the 95% CI does not include zero (MacKinnon, Lockwood, and Williams 2004). Model fit was evaluated using several indices, including the Chi‐squared (*χ* ^2^) goodness‐of‐fit test and its corresponding *p*‐value, the root mean square of approximation (RMSEA), standardized root mean squared residual (SRMR), and confirmatory fit index (CFI). Acceptable model fit is indicated by RMSEA ≤0.08, SRMR ≤0.08, and CFI ≥0.90 (Hu and Bentler 1998; MacCallum, Browne, and Sugawara 1996).

**TABLE 2:** Descriptive statistics and Pearson correlation matrix.

	*N*	Mean (SD)	Min–max	1	2	3	4	5
Prenatal								
Maternal DERS	288	77.39 (24.16)	36.00–155.00	—	—			
Maternal resting RSA	270	5.44 (1.19)	1.83–8.17	−0.05				
7 months								
Maternal everyday stress	219	9.51 (8.14)	0.00–45.00	0.33^**^	−0.13	—		
18 months								
Toddler expressive vocabulary	187	22.51 (19.92)	0.00–89.00	−0.06	0.19^*^	−0.21^**^	—	—
Toddler receptive vocabulary	187	62.34 (18.98)	6.00–89.00	0.06	0.13	0.01	0.51^**^	

Abbreviations: DERS, Difficulties in Emotion Regulation Scale; RSA, respiratory sinus arrhythmia; SD, standard deviation.

*p < 0.05; **p < 0.01.

## 3 Results

### 3.1 Preliminary Analyses

Descriptive statistics and correlations are presented in Table 2. A frequency distribution of the primary variables indicated that the data fell within a normal distribution (skewness ≤3 and kurtosis ≤10). Outliers (*N* = 9) were observed for the following variables: Maternal resting RSA (1 value less than 3 SDs below the mean), maternal DERS (2 values greater than 3 SDs above the mean), maternal everyday stress (4 values greater than 3 SDs above the mean), and toddler expressive vocabulary (2 values greater than 3 SDs above the mean). We conducted our analyses with the outliers included as is and with the outliers handled via Winsorization (Salkind, 2010). Because no significant differences emerged as a function of outlier management technique, we present our findings below with the outliers included to retain the natural variability present in the dataset.

### 3.2 Longitudinal Path Model

**FIGURE 1:**
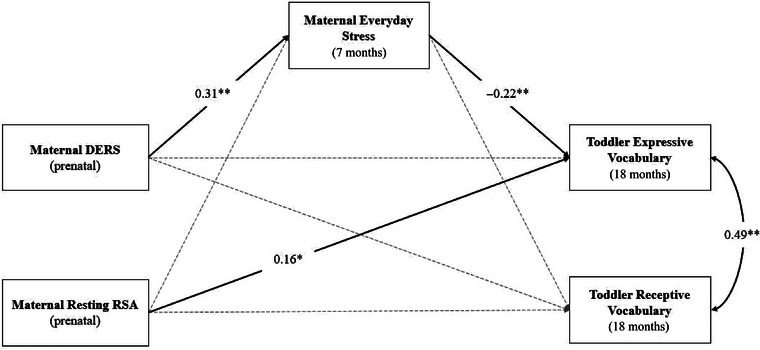
Path model examining the longitudinal relations between maternal prenatal emotion dysregulation, postnatal everyday stress, and toddler vocabulary. DERS, Difficulties in Emotion Regulation Scale; RSA, respiratory sinus arrhythmia. *p < 0.05, **p < 0.01. *N* = 289 for path analysis. Solid lines represent statistically significant pathways. Dashed lines represent nonsignificant paths. Standardized coefficients are presented. Covariates included child biological sex, child birth weight, and prenatal maternal education.

Path analysis was used to examine whether prenatal emotion dysregulation, measured via the DERS and resting RSA, was directly and indirectly related to toddler vocabulary outcomes (Figure 1). The model had acceptable fit: *χ* ^2^(8) = 13.71, *p* = 0.09; RMSEA = 0.05; SRMR = 0.05; CFI = 0.95. Expectant mothers’ DERS scores predicted postnatal stress, *β* = 0.31, *p* < 0.001, even after controlling for mothers’ educational status, *β* = −0.16, *p* = 0.03. Mothers who reported greater emotion dysregulation during their third trimester of pregnancy tended to also report experiencing greater postpartum everyday stress. By contrast, prenatal resting RSA did not significantly predict mothers’ postnatal stress scores, *β* = −0.10, *p* = 0.19. Together, the prenatal variables accounted for 13.4% of the variance in postnatal maternal everyday stress.

Even after controlling for child sex, *β* = −0.21, *p* = 0.01, prenatal resting RSA predicted toddlers’ *expressive* vocabulary skills at 18 months, *β* = 0.16, *p* = 0.03. That is, mothers with high resting RSA measured during pregnancy had children who produced more words during toddlerhood. Postnatal maternal everyday stress also predicted toddlers’ expressive vocabulary skills at 18 months, *β* = −0.22, *p* = 0.003. Thus, mothers who reported lower levels of postnatal everyday stress had children who produced more words during toddlerhood. Unexpectedly, the only significant predictors of toddlers’ *receptive* vocabulary skills were child sex, *β* = −0.27, *p* < 0.001, and child birth weight, *β* = 0.19, *p* = 0.01, whereby girls and children with higher birth weights exhibited a receptive vocabulary advantage in toddlerhood. Collectively, the estimated path model accounted for 12.4% and 9.2% of the total variance in toddler expressive and receptive vocabulary, respectively. The 95% bootstrap CIs were estimated to examine the hypothesized indirect relations between the prenatal emotion dysregulation variables and the toddler vocabulary outcomes at 18 months. There was a significant indirect effect of prenatal maternal DERS on toddler expressive vocabulary via postnatal maternal everyday stress, *β* = −0.07, SE = 0.03, *p* = 0.02, 95% CI [−0.147, −0.025].

### 3.3 Post Hoc Analysis

Although our approach to investigating whether prenatal maternal emotion dysregulation predicts postnatal stress was informed by developmental theory and prior research, we acknowledge that bidirectional associations between stress and mental health have been observed in the literature (Feng, Xu, and Lei 2023; Hammen 1991; Hammen et al. 2009; Levine, Milyavskaya, and Zuroff 2020), suggesting the possibility of an inverse pathway. Consequently, we conducted a post hoc analysis to explore whether prenatal maternal everyday stress predicts toddler vocabulary outcomes through maternal emotion regulation (i.e., DERS scores) measured at 7 months postpartum. This analysis was performed using a subset of the larger study sample (*N* = 138) as only the participants in Cohort 1 were administered the ESI during the prenatal visit. Poor model fit was achieved, *χ* ^2^(5) = 15.96, *p* = 0.007; RMSEA = 0.13; SRMR = 0.08; CFI = 0.73. Notably, prenatal maternal stress was not associated with postnatal maternal emotion dysregulation, *β* = 0.13, *p* = 0.20, nor did it predict toddler expressive vocabulary, *β* = −0.06, *p* = 0.63, or receptive vocabulary, *β* = 0.10, *p* = 0.40. Likewise, postnatal maternal emotion dysregulation was not associated with toddler expressive vocabulary, *β* = −0.12, *p* = 0.39, or receptive vocabulary, *β* = −0.23, *p* = 0.07. Additionally, we estimated a second post hoc model with household income included as a covariate to explore the potential role socioeconomic stressors associated with income may play in shaping postpartum stress and language development. In this model (*χ* ^2^(7) = 17.01, *p* = 0.02; RMSEA = 0.08; SRMR = 0.06; CFI = 0.89), income was not a significant predictor of maternal postpartum stress (*β* = 0.02, *p* = 0.83) nor child vocabulary (*β*_receptive_ = −0.05, *p* = 0.59; *β*_expressive_ = 0.10, *p* = 0.22). These findings suggest that these alternative models, although theoretically plausible, were a poor fit to the data.

## 4 Discussion

Our study builds upon prior perinatal and developmental research by examining associations between prenatal emotion dysregulation, postpartum maternal everyday stress, and toddler vocabulary development. We identified maternal resting RSA during the third trimester of pregnancy as a predictor of toddler expressive vocabulary skills at 18 months. Our findings also indicated that expectant mothers’ self‐reported emotion dysregulation was related to their perceived levels of postpartum everyday stress, which was in turn related to toddler vocabulary size. These results are further discussed below in light of previous literature.

Consistent with our hypothesis, our findings show that mothers who reported greater prenatal emotion dysregulation via the DERS also reported experiencing greater everyday stress postpartum. Previous research has established bidirectional relations between psychiatric diagnoses (e.g., depression) and everyday stress (Feng, Xu, and Lei 2023; Hammen 1991; Hammen et al. 2009; Levine, Milyavskaya, and Zuroff 2020) in adulthood. Thus, in line with the stress generation hypothesis (Hammen 1991), mothers who experience psychological distress may inadvertently perpetuate stressful environmental conditions, which then further contributes to both an increase in negative emotions and perceived stressors. Our findings replicate and extend this line of work by uncovering a positive association across the perinatal period between emotion dysregulation and perceived everyday stress. Furthermore, interventions during the prenatal period, specifically targeting emotion dysregulation, could aid mothers in their appraisal, perception of, and ability to cope with stressors. By enhancing regulation abilities, individuals may develop a repertoire of strategies to better navigate various stressors more effectively across the perinatal period, thereby reducing postpartum stress levels. Nonetheless, further longitudinal research is needed to evaluate how changes in emotion regulation during pregnancy may relate to fluctuations in maternal postpartum everyday stress among high‐ and low‐risk populations.

By contrast, we found that expectant mothers’ resting RSA did not predict their postnatal everyday stress scores. Contrary to our original hypothesis, these results suggest that various aspects of prenatal emotion dysregulation may have distinct associations with mothers’ self‐reported levels of postpartum everyday stress. It is important to note that while resting RSA is a reflection of an individual's capacity to respond to environmental demands (Beauchaine 2015; Porges 2007), RSA reactivity reflects the physiological changes that occur in response to environmental demands (Beauchaine and Thayor 2015; Porges 1995; Yaroslavsky et al. 2013). Thus, pregnant individuals’ RSA reactivity to emotional challenges may be a better predictor of later perceived everyday stress. Alternatively, it is also possible that prenatal resting RSA may be associated with postpartum everyday stress only among certain subpopulations of mothers, such as those experiencing adversity during pregnancy (e.g., low social support, limited access to prenatal care). According to diathesis–stress theories (e.g., Monroe and Simons 1991; Rosenthal 1963), psychological distress stems from a biological predisposition coupled with challenging environmental circumstances. Although investigations into maternal physiology × environment interaction effects were beyond the scope of the present study, further research examining the potential moderating role of risk and/or protective factors during pregnancy is warranted. Finally, the perinatal period represents a time during which expectant mothers undergo a significant increase in blood volume and numerous other physiological changes (e.g., cardiovascular, respiratory, endocrine; Soma‐Pillay et al. 2016), which may alter associations between maternal resting RSA and self‐reported psychological constructs during pregnancy. Consequently, resting RSA during the third trimester of pregnancy may be a weaker predictor of mothers’ postnatal everyday stress in comparison to measures of maternal postpartum RSA. In sum, future research should seek to incorporate an array of physiological indices measured across the perinatal period to better understand the potential longitudinal relations between maternal emotional dysregulation and everyday stress.

Next, we anticipated that both maternal prenatal emotion dysregulation and postnatal everyday stress would be directly related to children's vocabulary size during toddlerhood. We found mixed support for these hypotheses. First, we found that prenatal resting RSA (but not DERS score) was directly associated with toddlers’ *expressive* vocabulary skills at 18 months. In other words, pregnant mothers with high resting RSA, and therefore with a greater capacity for emotion regulation, had toddlers who produced more words during their second postnatal year. Situating these findings in the developmental literature is challenging given the dearth of research examining links between maternal physiology and toddler language acquisition; however, the direction of this association is consistent with existing literature linking maternal emotion dysregulation to child socioemotional development (Binion and Zalewski 2018; Ostlund et al. 2019), which is closely related to early language development (Beck et al. 2012). And yet, the nonsignificant, indirect pathway from maternal resting RSA to toddler vocabulary size via postpartum everyday stress would suggest that the mechanism underlying this relation remains unclear. Other aspects of the postnatal caregiving environment not directly measured in the current study may account for this association, such as maternal sensitivity (Baumwell, Tamis‐LeMonda, and Bornstein 1997) or maternal language input (Huttenlocher et al. 2010). However, it is important to note that language develops in the context of dynamic and contingent parent–child interactions (Kuhl 2007) and, therefore, child factors also play a role in predicting language development (e.g., temperament and neurobehavioral signs of stress; Bruce et al. 2022; Bruce et al. 2024; Noel, Peterson, and Jesso 2008). Indeed, environmental exposures during sensitive periods of fetal development can have lasting effects on offspring health (Barker 1998), and in utero stress exposure has been linked to adverse fetal and child neurocognitive development (Buss et al. 2011; Thomason et al. 2021; Nazzari et al. 2020). Thus, maternal RSA may be related to biologically based child factors, which may play a role in shaping language acquisition in infancy. Another promising avenue for future research is to examine early emerging child factors that may relate to both maternal RSA and child vocabulary development.

Second, our results show that mothers who reported lower everyday stress levels at 7 months postpartum had children who produced more words at 18 months (i.e., expressive vocabulary). It is well‐established that stress is related to maternal responsiveness and language input, which are in turn closely linked to toddler vocabulary acquisition (Hart and Risley 1992; Hoff‐Ginsberg 1986; Kuhl 2007). For instance, maternal stress is directly related to the quantity and quality of maternal speech (Noel, Peterson, and Jesso 2008; Pierce, Reilly, and Nelson 2021; Spinelli et al. 2022), in addition to mothers’ engagement in warm and responsive behaviors during playful linguistic exchanges with their toddler (Leigh, Nievar, and Nathans 2011; Steelman et al. 2002). Consistent with cognitive resource depletion theory (Feng, Xu, and Lei 2023; Padmala, Bauer, and Pessoa 2011), our study demonstrates that stressors perceived by mothers during the postpartum period, irrespective of their parenting‐specific nature, are predictive of toddlers; expressive vocabulary outcomes when measured the following year.

Furthermore, an indirect negative association was detected between prenatal maternal emotion dysregulation, as measured via the DERS, and toddler expressive vocabulary. That is, greater self‐reported difficulties with emotion regulation during pregnancy predicted greater postnatal everyday stress, which in turn predicted lower toddler expressive vocabulary abilities at 18 months. This novel finding further supports the downstream effect of prenatal emotion dysregulation on toddler development, and it identifies postpartum maternal everyday stress as a key risk pathway linking prenatal emotion dysregulation to early vocabulary acquisition. Pregnant individuals undergo significant neurobiological and psychosocial changes across the perinatal period, which can increase the likelihood of experiencing symptoms of depression and anxiety (Biaggi et al. 2016). Emerging evidence suggests that maternal emotion regulation can be improved during pregnancy, which has critical implications for maternal health and toddler development (Penner and Rutherford 2022). In accordance with existing research, our results illustrate the potential utility of early interventions bolstering maternal emotion regulation during pregnancy with respect to toddler expressive vocabulary acquisition.

Finally, the only significant predictors of toddlers’ receptive vocabulary skills were child sex and child birth weight. These covariates unexpectedly emerged as the sole predictors of children's receptive vocabulary skills, although it is important to acknowledge that this pattern of associations is consistent with the developmental literature (Bornstein, Hahn, and Haynes 2004; Madigan et al. 2015; Rinaldi et al. 2021). That is, female children have been shown to outperform males on measures of expressive and receptive language early in development, and low‐birth‐weight children, even those falling within a normative range, are at an increased risk for speech and language difficulties in early childhood. In considering the strength of these associations, it is possible that the perinatal maternal factors in our study did not account for sufficient variability in children's receptive vocabulary scores with these covariates included in the path model. However, it is also possible that maternal emotion dysregulation and everyday stress levels are differentially related to various aspects of early language development. That is, maternal stress may influence caregiver–child interactions in a way that enables passive listening yet disrupts opportunities for young children to practice and refine productive vocabulary skills. It is also worth noting that the developmental trajectory of expressive versus receptive vocabulary acquisition may play a role in shaping the relations between maternal stress, mental health, and child vocabulary over the course of early childhood. Indeed, children typically begin comprehending language prior to producing their first words (Fenson et al. 1994). It is therefore plausible that early expressive vocabulary deficits may diminish over time, particularly if children possess sufficient receptive vocabulary skills. Given the relative absence of literature examining these associations across the perinatal period, additional research is needed to build upon our study's findings. Specifically, future research should incorporate other measures of toddler language development (e.g., observational methods capturing lexical and grammatical knowledge) measured at multiple time points across infancy and early childhood.

Our results should be interpreted in light of several strengths and limitations. First, our study used a prospective longitudinal study design to better address novel questions regarding predictive associations between biobehavioral markers of maternal emotional dysregulation during pregnancy, everyday stress during the first postnatal year, and toddlers’ vocabulary acquisition. A notable strength of our study lies in its use of path analysis coupled with maximum likelihood estimation to handle missing cases, which enabled us to retain a large sample size of mother–toddler dyads and draw inferences using all available data. Furthermore, the current study is bolstered by its robust methodological and analytic approach, in addition to the large sample of mothers and toddlers recruited. Considerable effort was also made to recruit a sample of pregnant individuals from diverse demographic backgrounds and with a wide range of self‐reported emotion dysregulation scores. Nonetheless, our study's limitations present avenues for future research to expand upon our work. First, maternal report measures of expressive/receptive vocabulary may not capture the full breadth of children's emerging language abilities, and it is possible that more dysregulated mothers may be more likely to either under‐ or overestimate their child's verbal skills. Additional research is needed to explore how maternal mental health may shape maternal perceptions of child language and cognitive development. Ideally, behavioral and/or naturalistic language assessments can be incorporated into future studies to better understand the perinatal predictors of child language development more broadly. The current study also cannot speak to relations between prenatal emotion dysregulation, maternal everyday stress, and toddler language development among atypically developing or bilingual child samples, although replication research is certainly warranted within these populations. Finally, it is important to note that maternal stress is not a static construct as it likely varies across the postpartum period. Thus, future work should examine changes in maternal stress across the postpartum period in relation to toddler language acquisition.

## 5 Conclusions

Understanding the early antecedents to vocabulary development is essential to identifying evidence‐based targets for early intervention. The purpose of the present study was to examine longitudinal associations between prenatal emotion dysregulation, assessed via biobehavioral methods, and toddler expressive and receptive vocabulary at 18 months. Furthermore, we evaluated the role of maternal everyday stress at 7 months postpartum as it relates to maternal emotion dysregulation and toddler language acquisition. Our findings show that prenatal maternal emotion dysregulation, measured via self‐report and resting RSA, was differentially related to postpartum maternal everyday stress as well as children's expressive vocabulary outcomes. Taken together, our results highlight the potential importance of targeted interventions to promote maternal mental health during pregnancy, given downstream implications for maternal postpartum distress as well as toddler language development.

## Ethics Statement

This study was performed in line with the principles of the Declaration of Helsinki. Written informed consent was obtained from study participants. Approval was granted by the Institutional Review Board at the University of Utah.

## Conflicts of Interest

The authors declare no conflicts of interest.

## Data Availability

The data that support the findings of this study are available from the corresponding author upon reasonable request.
